# The Effect of West Nile Virus Infection on the Midgut Gene Expression of *Culex pipiens quinquefasciatus* Say (Diptera: Culicidae)

**DOI:** 10.3390/insects7040076

**Published:** 2016-12-19

**Authors:** Chelsea T. Smartt, Dongyoung Shin, Sheri L. Anderson

**Affiliations:** Florida Medical Entomology Laboratory, Department of Entomology and Nematology, University of Florida—IFAS, 200 9th Street Southeast, Vero Beach, FL 32962, USA; dshin@ufl.edu (D.S.); slanderson78@gmail.com (S.L.A.)

**Keywords:** gene expression, innate immunity, mosquito, gram negative bacteria binding protein, West Nile virus

## Abstract

The interaction of the mosquito and the invading virus is complex and can result in physiological and gene expression alterations in the insect. The association of West Nile virus (WNV) and *Culex pipiens quinquefasciatus* mosquitoes results in measurable changes in gene expression; 22 gene products were shown previously to have altered expression. Sequence analysis of one product, CQ G1A1, revealed 100% amino acid identity to gram negative bacteria binding proteins (CPQGBP) in *Cx. p. quinquefasciatus*, *Aedes aegypti* (70%) and *Anopheles gambiae* (63%) that function in pathogen recognition. CQ G1A1 also was differentially expressed following WNV infection in two populations of *Cx. p. quinquefasciatus* colonized from Florida with known differences in vector competence for WNV and showed spatial and temporal gene expression differences in midgut, thorax, and carcass tissues. These data suggest gene expression of CQ G1A1 is influenced by WNV infection and the WNV infection-controlled expression differs between populations and tissues.

## 1. Introduction

West Nile virus (WNV, family *Flaviviridae*, genus *Flavivirus*) is an important threat to humans and animals as it continues to cause morbidity and mortality in the United States [1] since its introduction into New York in 1999 [2]. The virus is maintained in an enzootic transmission cycle between birds and ornothophilic mosquitoes in the genus *Culex* [3]. *Culex pipiens pipiens* L., *Cx. p. quinquesfasciatus* Say, *Cx. tarsalis* Coquillett, and *Cx. nigripalpus* Theobald are all considered important vectors of WNV in the United States [4,5,6,7,8].

*Culex pipiens quinquefaciatus* is a vector whose competence for WNV varies between populations of mosquitoes [9,10]. Competence of a mosquito for a virus is influenced by both internal and external factors [11]. Viruses ingested with a blood meal must first infect midgut epithelial cells in order for the biological transmission of WNV to occur, and this represents the first barrier that the virus must overcome [11,12]. The barrier may be physical or due to the inactivation of virus by digestion enzymes [11]. Barriers to infection may be influenced by several different genes [13,14]. For example, two quantitative trait loci (QTL) influence a midgut infection barrier (MIB) to dengue virus-2 (DENV-2) in *Aedes aegytpi* (L.) [15,16] and these two QTL have been shown to be affiliated with vector competence [17].

Midgut gene expression can be influenced by the presence of virus in the blood meal [18,19,20]. Changes include altered levels of chitin-binding proteins, vesicle transporters, and components of the innate immune pathways [18,21,22]. Fluorescent differential display analyses showed several differentially expressed midgut genes in *Cx. p. quinquefasciatus* exposed to WNV compared to mosquitoes given an uninfected blood meal [20]. Several cDNAs (22) were altered in the presence of WNV. Temporal gene expression studies of one of the transcripts (CQ G12A2) with high similarity to a *Cx. p. quinquefasciatus* leucine-rich repeat-containing protein-like gene (LRR) showed that mRNA levels change in *Cx. p. quinquefaciatus* midguts that have been exposed to WNV compared to mosquitoes given uninfected blood meals. There were increases in CQ G12A2 (LRR) message after infection which corresponded to incubation periods in which WNV midgut titer was lowest, potentially implicating CQ G12A2 (LRR) in an immune response to WNV [20].

The objective of the study was to characterize selected genes in the midgut tissue of *Cx. p. quinquefasciatus* through sequence analysis and determination of gene expression changes after WNV exposure. In this study we describe the characterization of one gene, CQ G1A1 that was previously shown to be up-regulated in the *Cx. p. quinquefasciatus* midguts after exposure to WNV. Results from this study will contribute to the general understanding of the molecular interactions between the mosquito midgut and WNV that will improve our knowledge about mosquito biology and enhance our ability to control mosquito-borne disease.

## 2. Materials and Methods

### 2.1. Virus

Florida WNV isolate (WN-FL03p2-3) (GenBank accession number DQ983578) was passaged once in baby hamster kidney cells and four times in African green monkey kidney (Vero) cells prior to use. This strain is similar to the NY99 genotype through sequence analysis [23,24].

### 2.2. Mosquitoes

*Culex p. quinquefasciatus* from two different colonies were used. The first colony (CPQG) was established in 1995 from Gainesville, FL. The second colony (CPQV) was established in 2008 from Vero Beach, FL. Mosquitoes were reared at 28 °C under a 14:10 L:D cycle using standard methods [25,26]. Adult mosquitoes were provided 20% sugar solution and water ad libitum. Approximately 100–200 four to six day old mosquitoes were transferred to cardboard cages with mesh screening and sugar was removed from cages 24 h prior to each experiment.

### 2.3. Sequence Analysis

A differentially expressed PCR amplified product of interest (CQ G1A1) whose expression is up-regulated post WNV-infection was selected and cloned using TA cloning into the pCR2.1 cloning vector (TA cloning kit, Invitrogen, Carlsbad, CA, USA) [20]. Clones were prepared and sequenced following methods described by Smartt et al. (2009) [20]. BLAST and VectorBase analyses were used to find similarity between cloned sequences and sequences in GenBank using the published genome sequences of *Ae. aegypti* and *Cx. p. quinquefasciatus* [27,28].

### 2.4. Mosquito Infection, Tissue Dissection and RNA Extraction

Two *Culex p. quinquefasciatus* populations (CPQG and CPQV) were used to analyze CQ G1A1 expression differences after WNV infection. Only the CPQG population was used in the gene silencing experiment. For all experiments, one group was given a WNV-infected blood meal and the control group was given a blood meal without virus. Virus was propagated and blood meals were prepared using previously described methods [20,26].

Briefly, mosquitoes were allowed to feed for ca. 45 min on cotton pledgets soaked with defibrinated bovine blood (Hemostat, Dixon, CA, USA) and maintained at 28 °C for the duration of the experiment. Subsequent to feeding, mosquitoes were anesthetized with cold, fully engorged specimens, assessed visually, and transferred to new cages, and mosquitoes were provided 20% sugar solution as previously described [26]. RNA from samples was extracted as previously described using Trizol reagent [20]. Integrity of the RNA was determined using gel electrophoresis following standard procedures.

To determine involvement of WNV in the spatial expression of CQ G1A1, female mosquitoes from both mosquito populations were infected with WNV as mentioned previously and mosquito tissues, including midgut, carcass, thorax, and legs, were dissected at 4–16 hpi (hours post infection), 1–8 dpi (days post infection) for use in qPCR analysis. RNA from dissected midguts was extracted, as previously described.

To assess involvement of CQ G1A1 in WNV infection, three days post-inoculation of antisense molecules, CPQG mosquitoes in all treatment groups were fed citrated bovine blood and maintained as previously mentioned. Non-injected female mosquitoes of similar age were fed the same blood meals and samples collected at the same time points. Pooled samples of two to three midguts were dissected at different times post-infection: 4–16 hpi, 1–8 dpi for each treatment group. Dissected tissues were stored at −80 °C until further processing. RNA from dissected midgut tissues was extracted as previously described.

### 2.5. Generation of Antisense RNA and Microinjection of Mosquitoes

Gene silencing via dsRNA-mediated RNA interference was used to determine the extent to which the target antiviral genes [20] affect vector competence of *Cx. quinquefasciatus* for WNV. Silencing expression of the target genes was performed as follows: The MEGAScript T7 kit (Ambion, Austin, TX, USA) was used to generate dsRNA specific to the target genes, CQ G12A2 and CQ G1A1. The PCR products were quantified and 2 µg used as template for transcription under the control of the T7 promoter. After sequence verification of the PCR products, 0.5 µg of dsRNA diluted in 1 µL of distilled water was injected into the thoraxes of female mosquitoes. dsRNA was delivered into the hemolymph via the thorax of three-day old female *Cx. p. quinquefasciatus* using a Drummond Scientific Nanoject II injector (Thermo Fisher, Waltham, MA, USA). Mosquito treatment groups were as follows: (1) injected with either of the two target gene-specific dsRNA; (2) injected with dsRNA of a green fluorescent protein gene (GFP); (3) not injected; (4) injection of a mixture of dsRNA from both target genes. Approximately 95% of the injected mosquitoes survived using our methods. After inoculation, mosquitoes were transferred to incubators and maintained at 28 °C. Expression of CQ G12A2 (LRR-15 gene) in the RNAi samples will be presented and discussed in a follow on manuscript.

### 2.6. Semi-Quantitative RT-PCR and Quantitative RT-PCR

Semi-quantitative reverse transcription (RT)-PCR performed as previously described [20]. Integrity of the RNA was determined using gel electrophoresis following standard methods and RNA quantified on a NanoDrop 2000 Spectrophotometer (Thermo Fisher). Total RNA from each pooled sample (including uninjected and RNAi injected midgut samples with or without WNV) was treated with RQ1 RNase-free DNase (Promega, Madison, WI, USA) prior to RT-PCR to eliminate contamination from DNA. RT using the Enhanced Avian HS RT-PCR kit (Sigma Aldrich, St. Louis, MO, USA) was carried out following the included protocol. Semi-quantitative RT-PCR reactions were amplified on an MJ Mini Gradient Thermo Cycler (Bio-Rad Laboratories, Hercules, CA, USA). All RT-PCR products were analyzed on 2% agarose gels, stained with ethidium bromide, and visualized on an InGenius gel documentation system. The following primer sets were used to characterize gene expression (Integrated DNA Technologies, Coralville, IA, USA): CQ G1A1 forward primer, 5′-ACG AAG AGG GGA CTC ATC TGG GGG-3′, CQ G1A1 reverse primer, 5′-GGC AGC CAA TCG TCC CTT TTC TCC-3′. The CQ G1A1 primer set generated a 460 base pair (bp) product. Three replicates of semi-quantitative RT-PCR were performed for each time point. Expression of CQ G12A2 (LRR-15 gene) in the RNAi samples will be presented and discussed in a follow on manuscript.

Quantitative PCR of mosquito RNA was performed by reverse transcribing the RNA using Enhanced Avian Reverse Transcriptase (42 °C for 50 min, and qPCR reactions performed using SsoAdvanced SYBR green Supermix on a BioRad CFX96 Real-Time PCR Detection System following standard protocols (BioRad, Hercules, CA, USA). The qPCR conditions were 95 °C 30 s followed by 39 amplification cycles of 95 °C 5 s, 60 °C 30 s. The following primer set was used to characterize CQ G1A1 gene expression (Integrated DNA Technologies, Coralville, IA, USA): CQ G1A1 forward primer, 5′-ACG AAG AGG GGA CTC ATC TGG GGG-3′, CQ G1A1 reverse primer, 5′-GGC AGC CAA TCG TCC CTT TTC TCC-3′.

The quantity of WNV RNA in the blood meal and pooled tissue samples was determined using established methods [20]. All samples were tested two times in order to reduce sample errors. Standard curves were previously generated based on 10-fold serial dilutions of WNV determined by plaque assay [20,26].

### 2.7. Statistical Analysis

Virus titers in each pooled sample were determined from triplicate cycle threshold (Ct) values using Bio-Rad CFX manager software. Ct data was normalized by Log10 transformation and regression analysis used to determine a qPCR-derived titer (Qpfu/mL). The actin protein gene was used as an endogenous control gene and CQ G1A1 expression quantified with Real-Time quantitative RT-PCR.

## 3. Results

### 3.1. Sequence Analysis

The effect of WNV infection on the midgut gene expression of *Cx. p. quinquefasciatus* was studied using fluorescent differential display analysis [20]. One cDNA that was up-regulated in the presence of WNV, CQ G1A1 (accession no. JF907421), was 947 bp in length. Sequence analysis of this cDNA resulted in a putative translation product of 311 amino acids that was incomplete at the 5′ end. BLAST searches of the protein database with the putative translation product showed that it is 100% identical to a Gram-negative bacteria-binding protein (CPQGBP) in *Cx. p. quinquefasciatus* (GenBank accession no. XM_001845915.1; unpublished; Figure S1); 70% identical to CPQGBP in *Ae. aegypti* (accession no. XP_001659797; [28]); 63% identical to a protein in *An. gambiae* (accession no. XP_312118; [29,30]); and 54% identical to beta-1,3-glucan recognition protein 4 in *Bombyx mori* (accession no. NP_001159614; [31]). Using the conserved domain database (NCBI-Conserved Domain Database, [32]), the putative translation product of CQ G1A1 included a β-1,3 glucan binding domain, indicating it is related to a beta-1,3-glucan recognition protein family.

### 3.2. WNV Influences Temporal and Spatial Gene Expression

**Figure 1:**
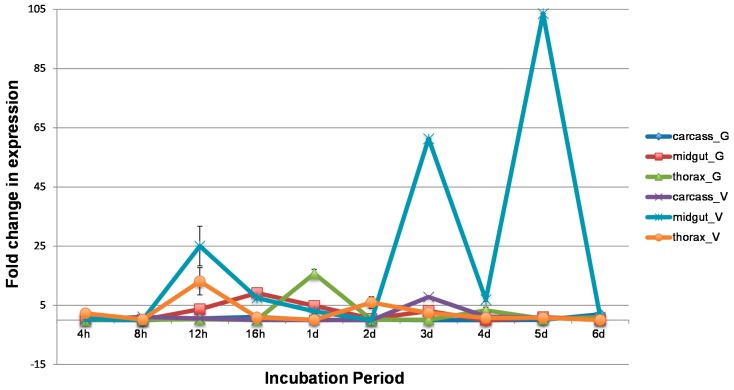
Fold change in CQ G1A1 gene expression in midgut tissue from two populations of *Cx. p. quinquefasciatus* (CPQG and CPQV) after West Nile virus (WNV) infection. G = CPQG (Gainesville); V = CPQV (Vero Beach); bp, base pairs; h, hours post infection; d, days post infection.

**Figure 2:**
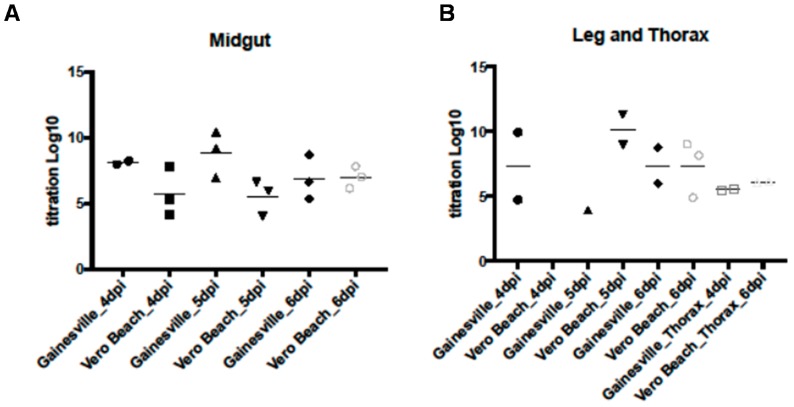
Titration of WNV in tissues dissected from two populations of *Cx. quinquefasciatus* (CPQG and CPQV) at different incubation periods after infection. (**A**) Titration of WNV from midgut tissues; (**B**) Titration of WNV from leg and thorax tissues. Gainesville = CPQG; Vero Beach = CPQV; dpi = days post infection.

The fold change in CQ G1A1 expression before and after infection with WNV was evaluated in the midgut, thorax, and carcass tissues dissected from two *Cx. p. quinquefasciatus* populations (CPQG and CPQV). A threefold or greater change in expression was detected in WNV infected midgut samples from the CPQG population at four time points, 12 hpi, 16 hpi, 1 dpi, and 3 dpi (Figure 1). The highest fold change in CQ G1A1 expression was at 16 hpi (Figure S2). WNV was detected in midgut tissue from the CPQG population at 12 hpi and 3 dpi but not at 16hpi, suggesting CQ G1A1 might be involved in an antiviral response as presence of WNV corresponded to low CQ G1A1 expression (fold change ≥ 3; Figure 1 and Figure 2). Higher fold change in expression of CQ G1A1 in midgut tissue from the CPQV population was found for most time points compared to CPQG, where fold change ranged from 103 to 6.9, with highest fold change at 3 dpi and 5 dpi (Figure 1). However, at 4 hpi, 8 hpi, 2 dpi, and 6 dpi there was little CQ G1A1 expression change between the treatment groups. WNV was detected at most time points, suggesting that CQ G1A1 expression in this population may not have a role in antiviral responses (Figure 2). Comparing CQ G1A1 expression between midgut tissues from both populations revealed only two time points with significant differences, 1 dpi and 3 dpi (*p* < 0.05). The large increases in CQ G1A1 expression in infected midgut tissue could suggest a role in WNV infection that is population specific. Greater than 3-fold change in expression was seen at 1 and 4 dpi in thorax from the CPQG population and detected in thorax from CPQV at 12 hpi and 2 dpi (Figure 1). WNV was not detected at these time points in either population but was detected by 6 dpi (Figure 2). WNV induced change in expression of CQ G1A1 > 3-fold was detected in carcass from the CPQV population (3 dpi) only. The change in expression in these tissues could indicate a role in other metabolic processes. There were few significant within population tissues-specific CQ G1A1 expression differences, which supports CQ G1A1 being ubiquitously expressed although WNV influences the expression.

### 3.3. WNV Titers in Tissues from Two Cx. quinquefasciatus Populations

WNV titer in tissues dissected from infected mosquitoes from both populations was assessed. WNV was detected in midgut tissue of CPQV population at most time points (Figure 2). In the CPQG population the titer of WNV was detected at 12 hpi and 3 dpi but was low, however, the average titer was above 6 log10 pfue/mL at 4 dpi and 5 dpi which is indicative of virus replication (Figure 2A). Overall, the CPQG population had higher midgut titers than the CPQV population. WNV was not detected in thorax tissue from either population until 6 dpi, and the titers were below 5 log10 pfue/mL (Figure 2B and Figure S2). Presence of WNV in leg tissues was detected from 4 dpi in both populations (data not shown), although titers did not increase above 7 log10 pfue/mL in the CPQG population while WNV titer in the CPQV population was highest titer at 5 dpi (≤10 log10 pfue/mL) (Figure 2B). Presence of WNV in midgut and leg tissue supports that both populations are competent vectors of WNV.

### 3.4. Suppression of Gene Expression Using RNAi

Gene expression in *Cx. quinquefasciatus* mosquitoes, both in midguts and whole bodies, is modulated by WNV [20,33]. We have shown expression variation in specific midgut genes following infection and the expression of some of these genes influences WNV replication, with the highest expression coinciding with a decrease in titer [20]. To investigate the role of CQ G1A1 in aspects of antiviral response, dsRNA for two cDNAs, CQ G12A2 [20] and CQ G1A1, were generated for microinjection. Female *Cx. quinquefasciatus* mosquitoes were injected with RNAi from either CQ G12A2, CQ G1A1, or a mixture of both.

**Figure 3:**
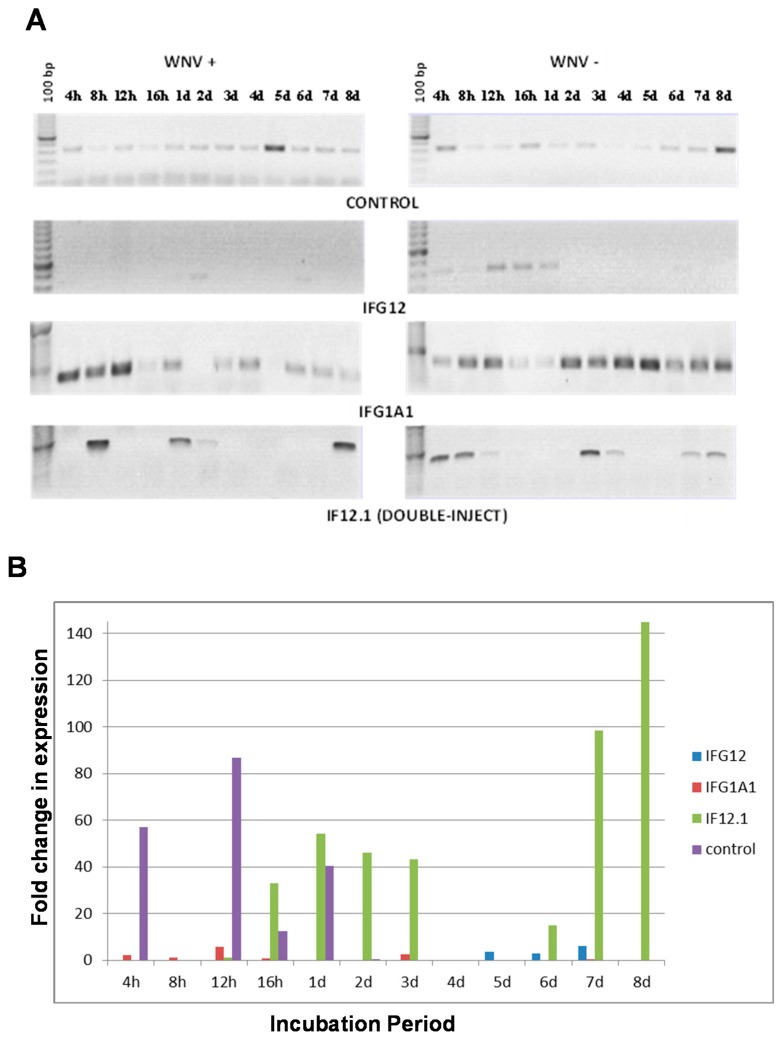
CQ G1A1 gene expression analysis in RNAi injected *Cx. quinquefasciatus* (CPQG) midguts dissected at different incubation periods post WNV infection. (**A**) RT-PCR analysis of CQ G1A1 expression; (**B**) qRT-PCR analysis of CQ G1A1 expression. 100 bp = DNA molecular weight marker; h, hours; d, days; Control, noninjected; IFG12, injected with CQ G12A2 RNAi; IFG1A1, injected with CQ G1A1 RNAi; IF12.1, injected with CQ G12A2 and CQ G1A1 RNAi. + = WNV in blood meal, − = no WNV in blood meal.

Semi quantitative RT-PCR of RNA from non-injected control samples showed low levels of CQ G1A1 expression in most samples regardless of infection status. The highest level of expression was detected on day 8 in uninfected midguts and 5 dpi for WNV infected midguts (Figure 3A). qPCR detection of changes in expression of CQ G1A1 in non-injected control samples (fold change ranging from ≥12–86) was seen only at early time points (4 h–1 day) with the highest at 12 h (Figure 3B). Surprisingly, suppression of CQ G12A2 expression limited the expression of CQ G1A1 in uninfected samples to the early time points compared to non-injected control samples (Figure 3A). Infection with WNV in the samples injected with CQ G12A2 RNAi resulted in little detectable CQ G1A1 product by semi-quantitative PCR, suggesting that expression of CQ G12A2 is essential for CQ G1A1 expression (Figure 3A); however, quantitative changes in CQ G1A1 expression were detected in these samples from 5 dpi to 7 dpi (fold change ≥ 2.9, Figure 3B). The absence of CQ G1A1 product upon silencing CQ G12A2 suggests that these two genes interact perhaps as members of the same pathway. Mosquitoes injected with RNAi to CQ G1A1 showed higher CQ G1A1 expression compared to non-injected controls, with expression mainly at later time points (2 day–8 day) then shifting to the early time points after WNV infection (4–12 h) (Figure 3A). WNV infection induced a fold change in expression (≥5) in CQ G1A1 injected samples at 12 h. In samples injected with both RNAi from both cDNAs, expression of CQ G1A1 was detected in midguts at only a few time points, however, assessment of fold change in expression revealed it was ≥32 at six time points (Figure 3B).

### 3.5. Effects of Silencing on WNV Replication

**Figure 4:**
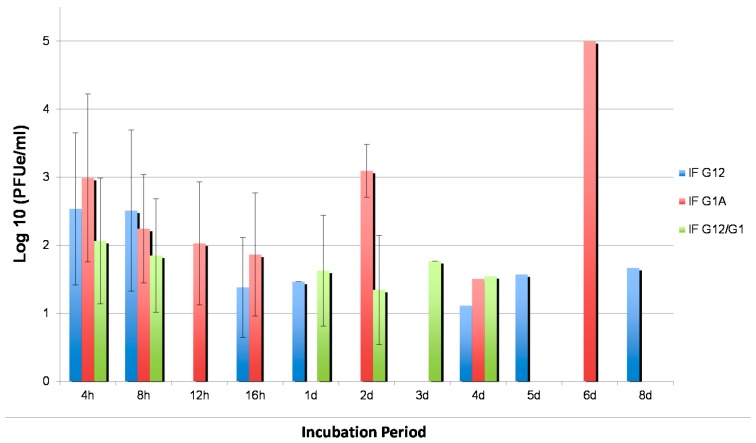
Titration of WNV in RNAi injected *Cx. quinquefasciatus* (CPQG) midguts dissected at different incubation periods post WNV infection. h, hours; d, days; IFG12, injected with CQ G12A2 RNAi; IFG1A1, injected with CQ G1A1 RNAi; IF12.1, injected with CQ G12A2 and CQ G1A1 RNAi.

WNV replication as a change in titer was evaluated in samples injected with antisense molecules and fed a WNV-containing blood meal to determine impact of silencing CQ G1A1 expression in female mosquitoes. For midgut samples injected with CQ G1A1 antisense, suppression resulted in high WNV replication (>3 log10 pfue, Figure 4) at 2 dpi and 6 dpi. WNV replication (>1.5 log10 pfue) was evident in midguts injected with CQ G1A1 RNAi in the early time points, when there were few changes in the expression of the gene between infected and uninfected samples. These results suggest that CQ G1A1 does not directly act as an antiviral molecule but may be involved in signaling activation of antiviral responses. Titer of WNV in samples injected with RNAi molecules to both cDNA was highest at 4 hpi but only reached about ≤2 log10 pfue; this represents a time during which expression of both cDNA was difficult to detect. In these samples, WNV replication was lowest at 2 dpi, which coincides with time points that have increased fold expression of CQ G1A1 following knockdown (Figure 3B and Figure 4). Overall, WNV titer was lower in midguts from double injected mosquitoes compared to those injected with CQ G1A1 alone, thereby supporting that CQ G1A1 is not directly involved in anti-WNV responses in these mosquitoes.

## 4. Discussion

The partial CQ G1A1 gene encodes a protein with similarity to the gram-negative bacteria binding protein family. The presence of a β-1,3 glucan binding domain as well as active sites similar to those found in the glycosyl hydrolase 16 family in the putative translation product of CQ G1A1 is support for the gene product having additional functions besides binding beta-1,3-glucanase, such as in pattern recognition [32,34]. The family of Gram-negative bacteria binding proteins (GNBPs) are pattern recognition receptors that can activate the Toll pathway in *Drosophila melanogaster* Meigen and *Ae. aegypti* [35] in response to Gram-positive bacteria, fungi, *Plasmodium* [36], and Gram-negative bacteria [37]. There are three subfamilies of GNBP. Subfamily B is specific to mosquitoes [38]. There are six members of the GNBP gene family (GNBPA1, GNBPA2, GNBPB1, GNBPB2, GNBPB3, GNBPB4) in *Anopheles gambiae* Giles, all of which are implicated in some aspects of immune response [39]. *Anopheles gambiae* GNBP has a β-1,3 glucan binding domain [40] and activates the mosquito Toll pathway as part of an immune response [35] to fungi, Gram-positive bacteria, *Plasmodium* [36], and Gram-negative bacteria [37]. The similarity of CQ G1A1 to *An. gambiae* GNBP-B is indicative that this gene could be involved in an immune response pathway such as the Toll-like pathway in response to bacteria [36]. The increase in G1A1 messages following WNV ingestion supports a potential association with an anti-WNV response [21,22]. The sequence identity between *Ae. aegypti* and *Cx. p. quinquefasciatus* GNBP was significant, but their functional characterization in these mosquitoes has yet to be performed.

Expression differences in two *Cx. p. quinquefasciatus* populations might be indicative of involvement in vector competence. *Cx. quinquefasciatus* from Gainesville infected with WNV revealed CQ G1A1 was expressed in midgut, thorax, and carcass tissues, although the highest fold change in expression occurred at early time points (Figure 1), suggesting that CQ G1A1 is a pattern recognition protein involved in WNV recognition. In the CPQV population expression change was most extreme in the midgut tissues following WNV infection, but occurred at later time points (i.e., after the virus has escaped the midgut [26,41]), suggesting involvement with processes beyond the recognition of WNV, perhaps related to the presence of bacteria in either blood or sugar meals [34]. Additionally, at the highest fold change in expression of G1A1 in midgut tissues, WNV is readily detected. CQ G1A1 expression in midgut tissue is significantly different between the two populations, and thus suggests a role in WNV infection that is population specific.

WNV was detected in midgut tissue of the CPQV population at most time points and in the CPQG population at later time points, and in both populations WNV titer increased with time indicative of virus replication [42,43]. Overall, the CPQG population had higher midgut titers than the CPQV population. Detection of WNV in midgut and leg tissue provides support for both populations being vectors of WNV [41].

Evaluation of the role of CQ G1A1 in WNV infection processes in midguts of the more WNV permissive *Cx. quinquefasciatus* population using gene expression silencing was performed. Surprisingly, injection with CQ G1A1 RNAi, increased G1A1 expression, and infection with WNV was shown to aid in suppression of gene expression later in the incubation period, thus changing temporal CQ G1A1 gene expression, under these conditions. This change in expression knockdown after infection could mean that WNV is able to interfere with the ability of this gene to be expressed [18]. Knockdown of CQ G12A2 resulted in little detectable CQ G1A1 product, suggesting that expression of CQ G12A2 is essential for CQ G1A1 expression. The absence of CQ G1A1 product upon silencing CQ G12A2 suggests that these two genes interact perhaps as members of the same pathway [39,44]. Samples that were silenced for both genes revealed changes in temporal CQ G1A1 expression that was also controlled by WNV. The change in expression knockdown efficiency after infection with WNV suggests the virus interfered with gene expression. The ability of a virus to control the expression of mosquito genes is not novel [18], and recent studies with dengue virus in *Aedes aegypti* have revealed the virus is repressing transcript enrichment in midgut tissue [45]. Although the titration of WNV in these RNAi injected midgut samples remained low, the presence of WNV was detected in all samples across time points, even samples where no CQ G1A1 expression could be detected, supporting a role for the CQ G1A1 gene product in functions beyond innate immune response to WNV [35].

Using fluorescent differential display analysis, two genes (i.e., CQ G12A2 [20] and CQ G1A1) in two immune response pathways (Toll and Imd) have been characterized with respect to WNV infection in *Cx. p. quinquefasciatus*. These results indicate that mosquito CQ G1A1 is constitutively expressed and its expression, although not directly linked to antiviral responses, alters after infection with WNV, and the increase in expression might be due to other factors, like presence of microbial cell wall components [35]. Expression differences in two *Cx. p. quinquefasciatus* populations might be indicative of involvement in vector competence. Because the two genes were expressed in the same differential display analysis and each product is suggested to be associated with a different arbovirus infection response pathway, there is additional support for the involvement of more than one immune pathway in the WNV infection process in these mosquitoes [46].

## 5. Conclusions

In this study the sequence of the CQ G1A1 gene showed high similarity to families of Gram-negative bacteria binding proteins which is support for CQ G1A1 being a member of proteins involved in pattern recognition. Results from the gene expression studies revealed CQ G1A1 expression is influenced by presence of WNV, is temporally and spatially differentially expressed, with midguts from the more WNV-competent mosquito population showing the highest level of expression, and is not solely associated with antiviral responses. The CQ G1A1 expression knockdown studies support that WNV is able to alter CQ G1A1 expression and reveal that CQ G1A1 expression is dependent on the presence of another transcript, CQ G12A2, also shown to be upregulated following WNV infection.

## Supplementary Materials

The following are available online at http://www.mdpi.com/2075-4450/7/4/76/s1, Figure S1: Multiple protein sequence alignment of a fragment of the *Culex pipiens quinquefasciatus* putative Gram-negative bacteria binding protein (CQ G1A1) with GNBP proteins from other insects, Figure S2: Titration of WNV in tissues dissected from two populations of *Cx. quinquefasciatus* (CPQG and CPQV) at different incubation periods after infection.
