# Supplementary material for: The Effect of West Nile Virus Infection on the Midgut Gene Expression of Culex pipiens quinquefasciatus Say (Diptera: Culicidae)
Source: Insects. 2016 Dec 19;7(4):76. doi: 10.3390/insects7040076 (PMC5198224; doi:10.3390/insects7040076)
Supplement: Supplementary file 1 [file insects-07-00076-s001.pdf]

Supplementary Materials: The Effect of West Nile Virus Infection on the Midgut Gene Expression of *Culex pipiens quinquefasciatus* Say (Diptera: Culicidae)

Chelsea T. Smartt, Dongyoung Shin and Sheri L. Anderson

```
CQ G1A1 partial protein    ---------------------------------------LNHRDNSFVEDGVLYLKP  18
CQ GNBP                    VDRKAFCPGELLFEDNFDTLDLDRTG--------------S..................  105
BM b1,3 glucan rec P-4      AP-VTVCSGQLIFADDFVDFDLEKWQHENTLAGGGNWEFQYYN.N.T...TNN.L..IR.  88
Ae GNBP                    VYRKTFCSGDLIFEDNFDKLNLEKWEHEHTLGGGGNWEFQYY..N.K..Y..N.I..IR.  104
An GNBP-B                  VKRSAFCPGDLIFEDNFDRLDLERWQHEVTLAGGGNWEFQYY..S.R....K..IFFIR.  87

CQ G1A1 partial protein    TFIGLEPGGEEYLKTGKLDINGGDPGNFCTNPAWDGCVRTGTPESILNPVKSARIRTAHS  78
CQ GNBP                    ............................................................  165
BM b1,3 glucan rec P-4      SLTSDQF.-SAF.HS.R.N.E..A.ADR....Q.Y..E.V...TN....I.......VN.  147
Ae GNBP                    .LLAD.T.-...F.TS.T.NL...S.YDS.........E......NP...I....L..LK.  163
An GNBP-B                  .LLAD.T.-...F.SS.N.NVH..T.YD.....SNY..E.Q.S.TNY...I....V..VN.  146

CQ G1A1 partial protein    FNFKYGKLEIRAKLPTGDWMWPALWLMPRTNQYGTWPASGEIDLMEARCNVDYRDEEGTH  138
CQ GNBP                    ............................................................  225
BM b1,3 glucan rec P-4      .S.Q...V.V...M.S...L...I....AY.K............V.S.G.KNMF-LN.L.  206
Ae GNBP                    ..................L........KL.......T........S.G.L...VAD...  223
An GNBP-B                  ...R...V.....I.....L........KI.......S........S.G.L..S-VN.NQ  205

CQ G1A1 partial protein    LGVEQVLSTLHFGPNAWTNAYDTSTAPKNSASGQGFNKDFHRYQLEWTPEFMKFSVDGEH  198
CQ GNBP                    ............................................................  285
BM b1,3 glucan rec P-4      I.TQEAG....Y..FPGLSGWERAHWVRRNSA.--YDTN..........D.IS.RI.DSE  264
Ae GNBP                    I.....G........PSL.GFE....A....P.E...N............Y......D.E  283
An GNBP-B                  ....H.GT......QWDL.G.EMA..V...PK......G............R....D.Q  265

CQ G1A1 partial protein    ILQVD---GNFWQRGNFDERAPGTRNPWVSGTKMAPFDQEFFIILNLAIGGTNGYFPDEK  255
CQ GNBP                    .....---....................................................  342
BM b1,3 glucan rec P-4      .GR.APGN.G..EY.G.NN.-...IH...RY.S.......K.YL.I...V.....F...G-  322
Ae GNBP                    T.V.----...............Q.....P...I..G......E..HV.M...V.........PP  340
An GNBP-B                  VM..E-------.....EL.R....R..VQ....T.G.........Y..M...V.....F...VP  322

CQ G1A1 partial protein    -VVN-TKPKPWSNQSPVGPAMTSFWEKRDDWLPTWNLDINDGKDAAFQIDYVRIWAL.  311
CQ GNBP                    ----......................................................  397
BM b1,3 glucan rec P-4      -.K.-PI....W.N..T--.A.D..NGQGG.......NV...Q..SL.V....V...  375
Ae GNBP                    -AT.KDN....T.G..T--.RGG..SAKE......K.EE..S.E.SL.V....V...  394
An GNBP-B                  PA..ANGN.....N..T--.LRD..LG.S......K.QE..SAE.S..V....V...  377
```

Figure S1. Multiple protein sequence alignment of a fragment of the *Culex pipiens quinquefasciatus* putative Gram-negative bacteria binding protein (CQ G1A1) with Gram-negative bacteria binding proteins (GNBPs) from other insects. Aligned protein sequences include the G1A1 putative translation product (311 amino acids; CQ G1A1 partial protein) GNBP from *Cx. p. quinquefasciatus*; (CQ GNBP, accession no. XP_001845967.1), β-1,3 glucan recognition Protein-4 from *Bombyx mori* (BM b1,3 glucan rec P-4, accession no. NP_001159614.1), GNBP from *Ae. aegypti* (Ae GNBP, accession no. XP_001659797.1), and GNBP subgroup B from *Anopheles gambiae* (An GNBP-B, accession no. XM_312118.3). The β-1,3-glucan binding domain is underlined. The dots represent amino acids that are the same as in the CQ G1A1 partial protein sequence, the numbering represents the amino acid number, the dashes are included to maximize spacing.

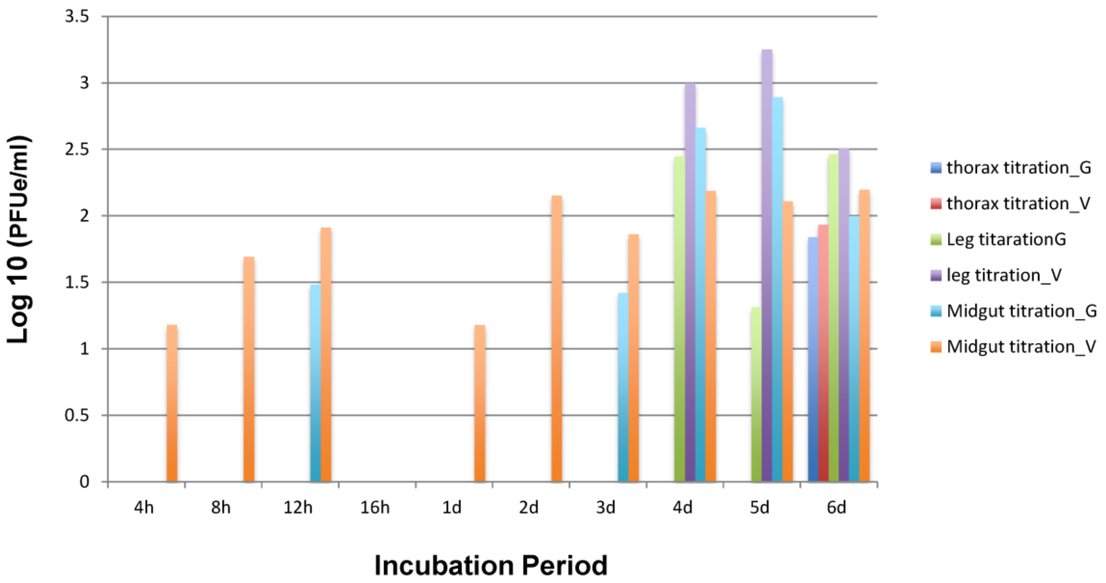

Figure S2. Titration of WNV in thorax, leg, and midgut tissues dissected from two populations of *Cx. quinquefasciatus* (CPQG and CPQV) at different incubation periods after infection. Gainesville = G; Vero Beach = V; h = hours post infection, d = days post infection.
